# Effect of Oxidative Stress on Diaphragm Dysfunction and Exercise Intervention in Chronic Obstructive Pulmonary Disease

**DOI:** 10.3389/fphys.2021.684453

**Published:** 2021-06-07

**Authors:** Bingzhi Zhang, Peijun Li, Jian Li, Xiaodan Liu, Weibing Wu

**Affiliations:** ^1^Department of Sports Medicine, Shanghai University of Sport, Shanghai, China; ^2^School of Rehabilitation Science, Shanghai University of Traditional Chinese Medicine, Shanghai, China

**Keywords:** COPD, exercise style, exercise intensity, oxidative stress, diaphragm dysfunction, exercise duration

## Abstract

Chronic obstructive pulmonary disease (COPD) can cause extrapulmonary injury such as diaphragm dysfunction. Oxidative stress is one of the main factors causing diaphragm dysfunction in COPD. Exercise plays a positive role in the prevention and treatment of diaphragm dysfunction in COPD, and the changes in diaphragm structure and function induced by exercise are closely related to the regulation of oxidative stress. Therefore, on the basis of the review of oxidative stress and the changes in diaphragm structure and function in COPD, this article analyzed the effects of exercise on oxidative stress and diaphragm dysfunction in COPD and explored the possible mechanism by which exercise improves oxidative stress. Studies have found that diaphragm dysfunction in COPD includes the decline of muscle strength, endurance, and activity. Oxidative stress mainly affects the structure and function of the diaphragm in COPD through protein oxidation, protease activation and calcium sensitivity reduction. The effects of exercise on oxidative stress level and diaphragm dysfunction may differ depending on the intensity, duration, and style of exercise. The mechanism of exercise on oxidative stress in the diaphragm of COPD may include improving antioxidant capacity, reducing oxidase activity and improving mitochondrial function.

## Introduction

Chronic obstructive pulmonary disease (COPD) is a chronic respiratory disease that progresses slowly. It is characterized by irreversible obstructive breathing. It is often related to smoking and can lead to chronic respiratory failure. With the aging of the world population and the high rates of smoking in Asia, COPD has become an increasingly serious problem in the twenty-first century (Raherison and Girodet, 2009; López-Campos et al., 2016). Respiratory muscle dysfunction is the main feature of acute and chronic respiratory failure in COPD. Dysfunction of the diaphragm, the main respiratory muscle, exists in all stages of COPD (Donaldson et al., 2012). Studies have shown that diaphragm dysfunction is associated with increased risk of hospitalization due to acute exacerbation of COPD, and inspiratory myasthenia due to diaphragm dysfunction is associated with dyspnea, hypercapnia, respiratory failure, and premature death (Caron et al., 2009; Vilaró et al., 2010).

Diaphragm dysfunction in COPD is mainly manifested in the structure and function, including sarcomere disruption, a loss of strength, and an increased susceptibility to failure in the face of a particular load (Orozco-Levi et al., 2001; Orozco-Levi, 2003). It is caused by different local and systemic factors. Pulmonary hyperinflation and increased work of breathing that occur in COPD are the main contributing factors to diaphragm dysfunction. In addition, systemic factors include inflammation, oxidative stress, hypoxia, and exacerbation. These factors can also influence the function of the diaphragm by inducing modifications in its local microenvironment. Oxidative stress affects the contractility and fatigability of the diaphragm and is one of the important mechanisms of diaphragm dysfunction in COPD (Langen et al., 2003; Gayan-Ramirez and Decramer, 2013; Gea et al., 2013). The structural changes caused by oxidative stress can weaken the strength of the diaphragm and promote muscle injury, and the increased production of free radicals in the diaphragm is related to respiratory failure caused by resistance breathing (Borzone et al., 1994; Klimathianaki et al., 2011). Therefore, studying the effects of oxidative stress on diaphragm function in COPD patients is beneficial to reduce the dysfunction and improve the quality of life and survival rate of these patients.

Exercise training can improve the function of the diaphragm possibly by improving the oxidative ability and efficiency of the skeletal muscle after exercise training, which decreases alveolar ventilation and dynamic hyperinflation, thus reducing diaphragm load (Nici et al., 2006). COPD is a systemic disease (Singh et al., 2019). Previous studies have found that inspiratory muscle training as a supplement to pulmonary function rehabilitation therapy can improve inspiratory muscle strength, but it does not provide additional benefits in exercise ability, quality of life, or dyspnea (Schultz et al., 2018). Therefore, compared with respiratory training, exercise training may have a better effect on the symptoms and function of COPD (Emtner and Wadell, 2016). Exercise training is undoubtedly the best choice to improve the muscle quality, function, and quality of life of patients with COPD (Emtner and Wadell, 2016; Paneroni et al., 2017). Previous studies have found that exercise training exerts a certain regulatory effect on the oxidative stress level of COPD diaphragm (Bowen et al., 2017a). However, the regulatory mechanism of exercise intervention on diaphragm dysfunction and oxidative stress in COPD patients remains unclear. Therefore, this study explored the relationship between oxidative stress and diaphragm dysfunction in COPD and reviewed the effects of exercise on oxidative stress in COPD diaphragm and its possible mechanism, so as to provide theoretical guidance and new ideas for exercise rehabilitation of diaphragm dysfunction in COPD.

## Diaphragm Dysfunction in COPD

In COPD, many different factors have beneficial or harmful effects on the structure and function of the diaphragm, and the positive adaptive changes of the diaphragm at least offset the influence of the latter to a certain extent (Gea et al., 2013). The following will discuss the performance of the diaphragm in COPD from two aspects of structure and function.

### Structural Changes of the Diaphragm in COPD

Among the positive adaptive changes of the diaphragm in COPD, the fiber type shifts toward type I fiber with higher oxidation degree. In previous studies, the proportion of slow-twitch fibers in the diaphragm was significantly higher than that in the control group, especially in moderate to severe patients (Levine et al., 1997; Ottenheijm et al., 2008; Barreiro et al., 2019). Endurance training increases the mitochondrial capacity of the diaphragm in COPD, and capillary proliferation is a late process in the adaptation (Ribera et al., 2003; Doucet et al., 2004). A shortening in the length of sarcomeres is another part of the positive adaptive change of the muscle (Orozco-Levi et al., 1999). Some of these adaptations appear to be proportional to the severity of airway obstruction, pulmonary hyperinflation, or air trapping in COPD. Thus, with increasing severity of COPD, these alterations may help the diaphragm cope with a higher workload associated with increased airflow limitation (Orozco-Levi et al., 1999; Wijnhoven et al., 2006).

Among the harmful effects of COPD on the diaphragm, muscle atrophy is one of the causes of diaphragm dysfunction. Studies have shown that in patients with severe COPD, the cross-sectional area of all types of muscle fibers is reduced by 40–60% compared with non-COPD controls and that apoptosis and atrophy of the diaphragm induced by myostatin overexpression can lead to diaphragm weakness and respiratory muscle dysfunction (Levine et al., 1997; Zhou et al., 2018). In addition, changes in lung morphology in COPD patients lead to diaphragm dysfunction. Chronic and dynamic hyperinflation forces the respiratory muscles to contract, leading to the shortening of the diaphragm far from its optimum length, which significantly affects its ability to produce force (Klimathianaki et al., 2011; Gayan-Ramirez and Decramer, 2013). The dome-like shape of the diaphragm is also crucial to its function. However, in patients with acute exacerbation of COPD, the lung is significantly affected by biomechanics, so it bears an increased load, lowering the diaphragm until it is almost flat between the chest and the abdomen (Bruells and Marx, 2018), which may change its shape. Finally, type I muscle fibers produce less force than type II muscle fibers, which may result in lower diaphragm muscle strength in mild to severe COPD patients than in healthy controls (Levine et al., 2003; Ottenheijm et al., 2005).

### Functional Changes of the Diaphragm in COPD

In terms of function, the increase of type I fibers, mitochondrial volume, and capillary number changes the energy metabolism of the diaphragm from glycolysis to oxidation (Gosker et al., 2000), resulting in the increase of aerobic capacity. Changes in the length of sarcomeres would partially counterbalance the negative effects induced by the displacement of the diaphragm length–tension curve on diaphragmatic force in patients with COPD (Gea et al., 2013). The adaptive mechanism mainly involves long-term exposure of the diaphragm to inspiratory load, making it necessary to remain active throughout the patient’s life, which will imitate muscle training (Barreiro and Gea, 2015). Therefore, the adaptive change of the diaphragm positively changes its strength and endurance. However, in COPD patients, the strength and endurance of the diaphragm often decrease despite experiencing positive adaptive changes that make it more resistant to fatigue. Atrophy, length, and shape changes decrease the strength and endurance of the diaphragm. In addition, diaphragm dysfunction in COPD also includes a lower diaphragmatic mobility (Gonçalves et al., 2018). Studies have shown that diaphragmatic mobility seems to be associated with airway obstruction, pulmonary hyperinflation, ventilatory capacity, and feeling of breathlessness (Rocha et al., 2017). Another study found that the reduction in diaphragmatic mobility in COPD patients was not influenced by respiratory muscle strength or pulmonary hyperinflation (Dos Santos Yamaguti et al., 2008). This may be caused by different sample sizes and different patient positions during testing. The loss of diaphragmatic mobility appears to be a decisive factor of decreased exercise tolerance and increased dyspnea in COPD patients (Paulin et al., 2007).

In stable COPD, diaphragmatic injury and adaptation achieve a special balance (Barreiro and Gea, 2015). From a clinical point of view, this special balance seems to provide sufficient lung ventilation for the survival of patients. However, the positive adaptive changes of the diaphragm are not sufficient to restore normal muscle strength and endurance; thus, this special balance leads to reduced diaphragm function, loss of muscle mass, and increased susceptibility to fatigue and injury (Orozco-Levi, 2003). Similowski et al. (1991) found that at the same lung volume, the diaphragm function of patients with stable COPD was equivalent to that of normal individuals, which may be because the compensatory phenomenon seems to counterbalance the harmful effects of hyperinflation on diaphragm contractility and inspiratory function. The predominance of factors such as exacerbations, nutritional abnormalities, and aging would make the balance tend to damage the diaphragm. For example, in advanced COPD, the harmful effects caused by injury, oxidative stress, and enhanced proteolysis will prevail over the beneficial effects, thus leading to respiratory failure and eventual death of the patients (Barreiro and Gea, 2015). During the period of acute-on-chronic hyperinflation in COPD, the ability of the diaphragm to generate transdiaphragmatic pressure is reduced, and these changes are exaggerated (Polkey et al., 1996).

In conclusion, there is a special balance between the damage and adaptive changes of diaphragm structure and function in COPD. Although the positive adaptive changes of the diaphragm in COPD can partially offset its dysfunction, the muscle strength, endurance, and activity of the diaphragm still show a downward trend. The mechanism of diaphragm injury is very complex, and oxidative stress is one of the important mechanisms.

## Oxidative Stress Promotes Diaphragm Dysfunction in COPD

The production of reactive oxygen species (ROS) is critical to diaphragm function. However, oxidative stress occurs when antioxidants are insufficient to resist the formation of free radicals (Heunks and Dekhuijzen, 2000). The mechanism of oxidative stress on diaphragm structure and function in COPD is very complex. Oxidative stress can activate diaphragmatic proteinase, increase protein oxidation, and decrease calcium sensitivity in COPD.

### Oxidative Stress Activates Diaphragmatic Protease in COPD

Oxidative stress is one of the factors that activate the protease pathway. The ubiquitin–proteasome system (UPS) is the main pathway of intracellular protein degradation. UPS plays a crucial role in the cascade that leads to the degradation of contractile proteins, thereby promoting the development of muscle atrophy, which constitutes the first step in the pathogenesis of diaphragmatic myasthenia (Ottenheijm et al., 2007; Debigaré et al., 2010). The activity of UPS in COPD diaphragm is enhanced (Ottenheijm et al., 2006), and oxidative stress may promote the activation of this pathway. Pomiès et al. (2016) showed that oxidative stress was involved in the *in vitro* atrophy of COPD skeletal muscle cells through the UPS signaling pathway. Mild oxidative stress can also increase protein degradation in the diaphragm by increasing the expression of the major components of UPS (Gomes-Marcondes and Tisdale, 2002). However, this pathway can only hydrolyze the peptide chain, not the complete myofibril. The calpain system can hydrolyze the skeletal protein in the myofibril, release the myofilament, and then activate UPS to play the role of protein hydrolysis (Liu et al., 2017). In COPD diaphragm, the expression of the calpain system is increased. Oxidative stress may increase the expression of calpain in the diaphragm, induce calpain activation, and then lead to atrophy of diaphragm fiber and dysfunction of contraction (Whidden et al., 2010). In conclusion, oxidative stress activates diaphragmatic proteinase in COPD, resulting in increased protein degradation, diaphragmatic atrophy, and dysfunction.

### Oxidative Stress Increases Diaphragmatic Protein Oxidation in COPD

Oxidative damaged proteins may misfold, and these misfolded proteins may form insoluble aggregates, posing a serious threat to cells (Kriegenburg et al., 2011). Barreiro et al. provided the first evidence of ROS and reactive nitrogen on oxidative modification of diaphragmatic protein in smokers and animals exposed to cigarette smoke for a long time. Oxidative damage of diaphragmatic protein may lead to muscle loss and dysfunction in smokers and COPD patients, and the level of diaphragmatic protein oxidation is significantly negatively correlated with muscle strength (Ottenheijm et al., 2008; Barreiro et al., 2010). In severe COPD patients, diaphragmatic protein oxidation, which is involved in energy production [creatine kinase (CK)] and contractile function [myosin heavy chain (MyHC)], may be part of the reasons for the decrease of diaphragmatic strength and diaphragm dysfunction (Marin-Corral et al., 2009). CK has been proved to be the main target of ROS exposure *in vitro* and *in vivo*. Oxidative modification of the muscle protein may have a negative impact on CK activity, leading to enzyme inactivation, and the oxidized protein is easier to be degraded, which may lead to muscle loss and dysfunction in smokers and COPD patients (Marin-Corral et al., 2009; Barreiro et al., 2010). MyHC is the basic unit of myosin, which plays an important role in ensuring the normal work of muscle cells. Carbonylation is the key to trigger the activation of the oxidation pathway (Carlos et al., 2014). A previous study found that the carbonylation degree of MyHC in the diaphragm of severe COPD was five times that of the healthy group, and the carbonylated MyHC experienced rapid degradation. The decrease of non-carbonylated MyHC could explain the decrease of the maximum transdiaphragmatic pressure and maximum inspiratory pressure in patients with severe COPD (Levine et al., 2013). In conclusion, oxidative stress in COPD diaphragm causes protein oxidation, the activity of the protein modified by oxidation is decreased, and the oxidized proteins can be easily degraded by protease, especially proteins with energy production and contraction functions, which leads to the dysfunction of COPD diaphragm.

### Oxidative Stress Reduces Diaphragmatic Calcium Sensitivity in COPD

In COPD patients, a single fiber is less sensitive to calcium produced by force and slows down the cross-bridge cycling kinetics, which may lead to muscle weakness during submaximal activation (Ottenheijm et al., 2005). Studies have found that excessive production of free radicals is related to impaired contractility. Long-term oxidative stress can increase the level of cytoplasmic calcium ions and reduce the production of diaphragmatic muscle strength, which indicates that oxidative stress reduces the sensitivity of diaphragmatic muscle fibers to Ca^2+^ (Heunks and Dekhuijzen, 2000). As a kind of reactive nitrogen, nitric oxide can reduce Ca^2+^ sensitivity by reducing the number of cross bridges in the strongly bound state and weaken cross-bridge cycling kinetics during submaximal activation (Heunks et al., 2001). Ca^2+^ binding with troponin induces a series of protein structural changes and ATP hydrolysis to release energy, which plays a key role in the process of muscle contraction. Therefore, the decrease of Ca^2+^ sensitivity will affect the contraction process of the diaphragm. In addition, decreased Ca^2+^ sensitivity requires higher cytosolic calcium to maintain equivalent force generation, resulting in increased consumption of ATP by Ca^2+^-ATPase. At the set submaximal activation, decreased Ca^2+^ sensitivity may affect the production of diaphragmatic force *in vivo* (Ottenheijm et al., 2007). These results suggest that oxidative stress may affect diaphragmatic muscle strength in COPD by reducing Ca^2+^ sensitivity.

In conclusion, oxidative stress can make diaphragm protein more easily degraded by activating proteases and oxidizing proteins, and the damage to oxidized proteins will also impair its function. In addition, oxidative stress can reduce the sensitivity of diaphragm fiber to Ca^2+^, which has an important impact on the structure and function of the diaphragm in COPD patients. Therefore, the regulation of the oxidative stress level in COPD diaphragm may be an important target to improve the structure and function of COPD diaphragm and prevent the further development of the disease.

## Effect and Mechanism of Exercise on Oxidative Stress of the Diaphragm in COPD

### Effect of Exercise on Oxidative Stress of the Diaphragm in COPD

Exercise training is an important part of pulmonary rehabilitation (Spruit et al., 2013; Gimeno-Santos et al., 2014) and can regulate the oxidative stress level of the diaphragm in COPD; however, the effects of exercise may differ depending on the intensity, duration, and style of exercise.

#### Effect of Different Exercise Intensities on Oxidative Stress of the Diaphragm in COPD

Exercise intensity is the core component of exercise prescription, and appropriate exercise intensity is very important for the recovery of COPD patients. Vieira Ramos et al. (2018) conducted moderate-intensity (50% of maximal speed) aerobic training on mice with COPD for 24 weeks and found that it upregulated the antioxidant gene of the diaphragm, controlled the decrease in diaphragm muscle mass in mice with COPD, and improved diaphragm atrophy caused by cigarette smoke exposure. However, the 2007 American Pulmonary Rehabilitation Guidelines indicated that high-intensity training may produce better physiological training effects, including reduced ventilation per minute and heart rate, thereby reducing dyspnea during submaximal exercise (Ries et al., 2007). The high-intensity training here refers to the intensity close to the individual’s peak level, which is a kind of relatively high intensity and is operationally defined as achieving at least 60–80% peak working speed during incremental maximum exercise test, as opposed to absolute high-intensity exercise. On the contrary, a single session of strenuous exercise may increase the oxidative stress level of the diaphragm in COPD patients, leading to diaphragm injury, dysfunction, or fatigue. This is because COPD patients may be in a state of calcium deficiency, and strenuous exercise enhances the calcium-restricted induced oxidative stress (Itoh et al., 2004). Studies have found that moderate-intensity (50–80% VO_2m__*ax*_) rather than high-intensity training has a beneficial effect on oxidative stress in elderly patients (Bouzid et al., 2015). This result may be due to reduced ventilatory capacity, abnormal gas exchange, and skeletal muscle dysfunction in elderly patients with COPD, which affect their performance in exercise and reduce their exercise ability and maximum work rate (Lopes et al., 2018). Moreover, strenuous exercise depleted muscle antioxidant vitamin levels, which may impair overall antioxidant protection (Ji et al., 1998). Therefore, in the exercise training of COPD, high-intensity aerobic exercise within the maximum peak exercise level may be more conducive to the regulation of oxidative stress level and functional recovery of the diaphragm in COPD than moderate-intensity exercise; however, elderly patients may be more suitable for moderate-intensity exercise than high-intensity exercise, and strenuous exercise should be avoided.

#### Effects of Different Exercise Durations on Oxidative Stress of the Diaphragm in COPD

A previous study found that 24 weeks of treadmill aerobic training improved the oxidative stress level of the diaphragm in mice with COPD (Vieira Ramos et al., 2018). Six weeks of high-intensity intermittent exercise (HIIT) attenuated oxidative stress in the diaphragm of smoke-exposed mice (Bowen et al., 2017a). In addition, many studies have found that long-term (≥4 weeks) aerobic training can improve the activity of antioxidant enzymes and the resistance of the diaphragm to intracellular ROS, reduce the oxidative modification of key proteins and enzymes, improve oxidative stress level of the diaphragm, and prevent diaphragm dysfunction (Oh-ishi et al., 1997; Mangner et al., 2013, 2016). However, other studies have found that short-term exercise training can also improve oxidative stress of the diaphragm. Vincent et al. (2000) found that 5 days of 65% VO_2m__*ax*_ short-term endurance exercise training can protect rat diaphragm from oxidative stress induced by contraction and reduce lipid damage induced by oxidation after long-term contraction. Smuder et al. (2012) found that 10 days of endurance exercise with 70% maximal oxygen consumption can protect rat diaphragm from mitochondrial oxidative damage induced by mechanical ventilation. In addition, Bowen et al. (2017b) found that 2-week HIIT reduced oxidative stress in the diaphragm and directly prevented oxidant-mediated diaphragm dysfunction in hypertensive mice. In conclusion, long-term aerobic exercise (≥ 4 weeks) seems to have a better regulatory effect on oxidative stress level of the diaphragm in COPD. As a more time-saving option, short-term exercise training can improve the oxidative stress level of the diaphragm, and its effect on oxidative stress level and function of the diaphragm in COPD is worthy of further study. Future studies should investigate the effects of different exercise durations on oxidative stress and function of the diaphragm in COPD, so as to determine the best exercise prescription with the best effect and the least cost.

#### Effects of Different Exercise Styles on Oxidative Stress of the Diaphragm in COPD

Many studies have found that aerobic exercise can improve the oxidative stress level of the diaphragm in COPD. Vieira Ramos et al. (2018) conducted treadmill aerobic training on mice with COPD and found that it improved the antioxidant capacity of the diaphragm. Li et al. (2020) found that after aerobic exercise in water, the nicotinic acid metabolism level in the diaphragm of COPD rats was upregulated, thus protecting the diaphragm and ventilatory muscle from oxidative damage and improving diaphragm muscle strength and ventilatory function. However, this experiment did not directly detect oxidative stress indicators. HIIT is a kind of aerobic exercise. Bowen et al. (2017a) found that HIIT attenuated oxidative stress of the diaphragm in smoke-exposed mice and increased diaphragm muscle strength. In addition, some studies found that resistance exercise (a kind of anaerobic exercise) can reduce serum lipid peroxidation and provide protection against oxidants (Vincent et al., 2002). Alcazar et al. (2019) found that the combination of HIIT and strength training can improve blood protein carbonylation and systemic oxidative stress in elderly COPD patients. In conclusion, different forms of aerobic exercise (water, land, and HIIT) have a good regulatory effect on the oxidative stress level of COPD diaphragm. Resistance exercise and combined exercise (resistance exercise and endurance exercise) can also affect systemic oxidative stress in COPD, but their effects on diaphragm function and oxidative stress level are still unknown and need to be further studied. The related literature of exercise intervention on oxidative stress of the diaphragm is shown in Table 1.

**TABLE 1:** Literature study on the effect of exercise on oxidative stress of diaphragm.

**Study**	**Research objects**	**Group**	**N**	**Exercise style**	**Exercise intensity**	**Exercise duration/frequency**	**Oxidative stress indicators**	**Diaphragm function indicators**
Vieira Ramos et al. (2018)	COPD mice	Exercise + Smoke group Smoke group	8/8	Treadmill aerobic training	50% Wmax	24 weeks, 5 days/week, 60 min/day	NRF2↑, Hmox1↑, Keap1↑	Muscle Mass↑ Atrophy↓
Bowen et al. (2017a)	Smoke exposed mice	HIIT + Smoke group Smoke group	11/10	HIIT	25°incline, 90% RW_*peak*_ + 60% RW_*peak*_	6 weeks, 5 days/week, 1 h(10 times, 4 min + 2 min interval)	NOX↓, SOD, CAT	Muscle strength↑
Heunks et al. (2000)	Emphysematous hamster	Exercise group Sedentary group	15/17	Treadmill training	5°incline, First week: 11.4 m/min; 2–6 weeks: gradually reach 20 m/min; 7–12 weeks: 20 m/min	12 weeks, 5 days/week, First week: 40 min/day; 2–6 weeks: gradually reach 60 min/day; 7–12 weeks: 60 min/day	GSH, GSSG, GSSG/GSH	Maximal tetanic force↑
Oh-ishi et al. (1997)	Male wistar rats	Training group Untrained group	14/14	Treadmill training	8°incline, First week: 15 m/min, gradually increase, 30 m/min after 6–7 weeks	9 weeks, 5 days/week, First week: 20 min/day, gradually increase, 90 min/day after 6–7 weeks	Mn-SOD↑, Cu, Zn-SOD↑, GPX↑, CAT↑	Endurance↑
Bowen et al. (2017b)	Hypertensive mice	DOCA-SALT + HIIT group DOCA-SALT group Sham group	15/11/11	HIIT	25°incline, 90% RW_*peak*_ + 60% RW_*peak*_	2 weeks, 8 times in total, 28 min (4 times, 4 min + 3 min interval)	NOX↓, SOD↑, CAT, GPX, Carbonylated MyHC↓	Muscle strength↑
Mangner et al. (2013)	Female C57BL6 mice	Exercise group Sedentary group	20/20	Treadmill training	5°incline, 15 m/min	4 weeks, 5 days/week, rest for 2 min every 15 min, 60 min/day	GPX↑, CAT, SOD, Mn-SOD, NOX, XO↓, Aconitase/Fumarase activity, Carbonylated α-Actin, Carbonylated CK	Muscle strength
Vincent et al. (2000)	Male SD rats	Exercise group Sedentary group	14/14	Treadmill training	0°incline, 65%VO_2m__*ax*_, 25 m/min	5 days, First day: 40 min; Day 2: 50 min; Days 3, 4 and 5: 60 min	CS↑, CAT↑, SOD↑, GPX, Lipid H_2_O_2_↓, GSH↓	Endurance↑ Fatigue resistance↑
Nakatani et al. (2005)	Male wistar rats	Habitual exercise group Sedentary group	19/16	Swimming	1–5 weeks: No load 6–10 weeks: 5% of body weight	10 weeks, 5 days/week, 15 min/day at the beginning, gradually increased to 60 min/day in the 7th week	SOD↑, CAT, GPX	/
Mangner et al. (2016)	HF mice	HF group HF + AET group	10/10	Treadmill training	15°incline, 15 m/min	9 weeks, 5 days/week, 1 h/day	XO↓, SOD, GPX, CAT↓, Carbonylated Action↓, Carbonylated CK↓	Muscle strength↑
Smuder et al. (2012)	MV rats	MV + Sedentary control group MV + Endurance training group Anesthetized sedentary control group	8/8/8	Treadmill training	0°incline, 30 m/min (70% VO_2m__*ax*_)	10 days, 60 min/day, 2 days off	H_2_O_2_, 4-HNE/protein adducts↓, GPX1↑, SOD1↑, SOD2↑	Contractile dysfunction↓ Atrophy↓

*/:There is no relevant expression in this paper ↓:The data of the experimental group was significantly lower than that of the control group ↑:The data of the experimental group was significantly higher than that of the control group.*

*NRF2, nuclear factor E2-related factor 2; Hmox1, heme oxygenase 1; Keap1, kelch-like ECH-associated protein 1; NOX, NADPH oxidase; SOD, superoxide dismutase; CAT, catalase; GSH, glutathione; GSSG, oxidized glutathione; H_2_O_2_, hydrogen peroxide; GPX, glutathione peroxidase; MyHC, myosin heavy chain; XO, xanthine oxidase; CK, creatine kinase; CS, citrate synthase; VO_2m__*ax*_, maximal oxygen uptake; RWpeak, peak power load; Wmax, maximum exercise load; DOCA, deoxycorticosterone acetate; HF, heart failure; AET, aerobic training; MV, mechanical ventilation.*

### Mechanism of Exercise on Oxidative Stress of the Diaphragm in COPD

#### Exercise Improves Antioxidant Capacity

Exercise training can accelerate the clearance of free radicals, reduce oxidative modification and injury, regulate the level of oxidative stress, and improve diaphragm function through the activation of antioxidants in the diaphragm. Antioxidants in the body include enzymatic and non-enzymatic substances. The antioxidant enzymes, including heme oxygenase-1 (HO-1), quinone oxidoreductase 1, catalase (CAT), glutathione peroxidase (GPX), and superoxide dismutase (SOD), are regulated by the nuclear factor-erythroid 2-related factor 2 (NRF2) pathway (Li and Duan, 2011). Studies have shown that exercise can upregulate the expression of HO-1 by upregulating the NRF2/HO-1 antioxidant pathway (Vieira Ramos et al., 2018), which can regulate the balance of oxidation and antioxidants, protect the diaphragm from atrophy caused by cigarette smoke exposure, and improve diaphragm dysfunction. SOD, GPX, and CAT are considered to be the first line of defense against oxidative stress *in vivo* (Powers and Jackson, 2008). Studies have found that exercise can lead to exercise-induced oxidative stress, but endurance training can improve the activities of antioxidant enzymes (Mn-SOD, Cu, Zn-SOD, GPX, and CAT), which may improve the resistance of rats to intracellular ROS, so as to protect the diaphragm from oxidative damage (Oh-ishi et al., 1997). However, the effect of exercise on the antioxidant capacity of the diaphragm remains to be fully elucidated. Nakatani et al. (2005) found that habitual exercise increased SOD activity in the rat diaphragm, but no evidence showed that it increased the activity of CAT or GPX. On the contrary, acute exercise can improve the activities of GPX and CAT in the diaphragm of untrained rats, but has no significant effect on the activities of Mn-SOD and Cu, Zn-SOD (Oh-ishi et al., 1997). Another study showed that exercise training increased the protein abundance of SOD1, GPX1, and CAT in the cytosol of rat diaphragm fiber, whereas only SOD2 and GPX1 were increased in the mitochondria of the diaphragm (Smuder et al., 2012). The differences in the test results may be caused by different exercise interventions, different diseases and physiological states of rats, or different diaphragm test sites. In addition, glutathione has an antioxidant effect; however, endurance training had no significant effect on the diaphragmatic glutathione in hamsters with emphysema (Heunks et al., 2000). In conclusion, exercise training may reduce oxidative damage by improving the antioxidant capacity of the diaphragm and improve the atrophy and dysfunction of the diaphragm in COPD, but the specific mechanism still needs to be further studied.

#### Exercise Reduces Oxidase Activity

Exercise can regulate the oxidative stress level of the diaphragm in COPD by reducing the activity of oxidase. NADPH oxidase (NOX) is the main oxidase. NOX, a membrane-bound complex, is a complex enzyme system found in phagocytes and epithelial cells. At the same time, NOX is the main enzyme generating reactive oxygen. Compared with non-smokers, the number of neutrophils and macrophages migrating to smokers’ lungs is increased, and ROS can be produced through the NOX system (Macnee, 2001), leading to oxidative stress of the diaphragm in COPD patients. Bowen et al. (2017a) found that exercise training can reduce the activity of NOX in the diaphragm of mice with COPD, regulate the oxidative stress level, weaken protein degradation, reverse the extrapulmonary damage to the diaphragm caused by smoking, enhance the muscle strength of the diaphragm, and restore the diaphragm function. In addition, the study found that the oxidative stress produced by NOX may be closely related to the expression level of pro-inflammatory factors such as tumor necrosis factor (TNF), and the antioxidant effect was partly responsible for the decrease of the expression level of pro-inflammatory factors (Wieczfinska et al., 2019). Blockage of TNF-α can significantly reduce the number of inflammatory cells and the production of superoxide anion in the diaphragm and improve the structure and function of the diaphragm (Domínguez-Álvarez et al., 2014). Therefore, exercise can not only reduce the production of ROS directly, but also reduce the production of ROS by reducing the expression level of pro-inflammatory factors, so as to regulate the oxidative stress level and improve the diaphragm structure and dysfunction in COPD.

#### Exercise Improves Mitochondrial Function

**FIGURE 1:**
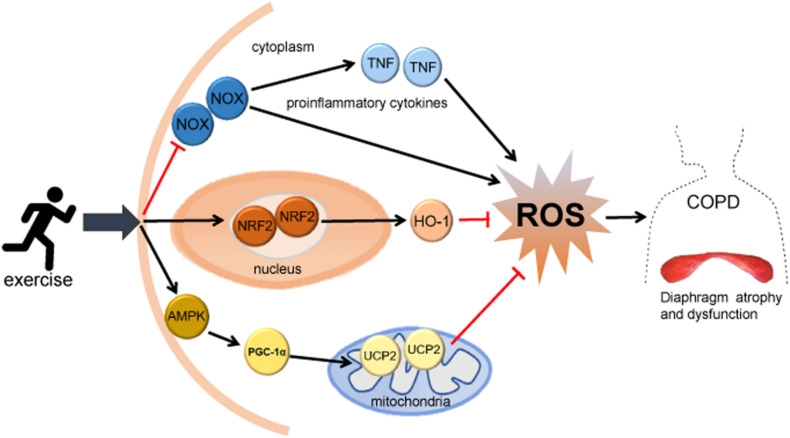
The mechanism of exercise on oxidative stress of the diaphragm in COPD. Exercise may reduce ROS production, regulate diaphragm oxidative stress level, and improve diaphragm structure and dysfunction in COPD through: (1) activating the NRF2 signaling pathway and upregulating HO-1 and other antioxidant enzymes; (2) inhibiting the activity of NOX and reducing the expression of TNF; and (3) activating the mitochondrial-related AMPK/PGC-1α/UCP2 pathway. HO-1, heme oxygenase-1; NOX, NADPH oxidase; TNF, tumor necrosis factor; ROS, reactive oxygen species; AMPK, AMP-activated protein kinase; PGC-1α, peroxisome proliferator-activated receptor gamma coactivator-1α; UCP2, uncoupling protein 2.

Exercise can regulate the oxidative stress level of the diaphragm in COPD by improving mitochondrial function. Mitochondrial dysfunction is a trigger factor of COPD and other diseases related to respiratory aging. Mitochondrion is one of the main endogenous sources of ROS, and its damage may increase the electron leakage of the electron transport chain and the formation of ROS, causing oxidative stress (Białas et al., 2016; Fang et al., 2019). Mitochondria of the diaphragm of rats exposed to cigarette smoke for a long time are damaged, showing fuzzy Z disk, unclear structure and focal myofilament rupture and dissolution (Sheng et al., 2020), which lead to the increased production of ROS. AMP-activated protein kinase (AMPK) has specific regulation on all aspects of mitochondrial biology and homeostasis, and exercise training can improve mitochondrial function by activating the AMPK pathway (Herzig and Shaw, 2018). Recent studies have found that the production of mitochondrial ROS is closely related to the AMPK pathway, which can limit the production of ROS, participate in the clearance of mitochondrial ROS, and increase the quality and function of the mitochondria by upregulating the expression of mitochondrial uncoupling protein (UCP) (Xie et al., 2008; Rubattu et al., 2016; Hasan-Olive et al., 2019). Peroxisome proliferator-activated receptor γ coactivator 1 (PGC1) is the downstream effector of AMPK, and most genes involved in mitochondrial metabolism are controlled by the PGC1 family (Herzig and Shaw, 2018). UCP2 is a cationic carrier protein on mitochondrial inner membrane, which can inhibit the production of ROS and protect mitochondrial function. It is also considered to be a direct target of PGC-1α transcriptional regulation (Jia, 2011; Gu et al., 2014). Studies have found that exercise training can upregulate the expression level of AMPK in skeletal muscle and reduce the production of mitochondrial ROS by upregulating the AMPK/PGC-1α/UCP2 pathway (Gu et al., 2014; Lin et al., 2020). Therefore, exercise may improve mitochondrial function and reduce ROS production by upregulating the AMPK/PGC-1α/UCP2 pathway, thereby regulating oxidative stress and improving dysfunction in the diaphragm of COPD. The mechanism of exercise on oxidative stress of the diaphragm in COPD is shown in Figure 1.

## Conclusion

The structure and function of the diaphragm in COPD are impaired and adaptive. In the stable period, the positive adaptive changes of the diaphragm can partially offset its dysfunction and achieve a special balance to meet the patient’s ventilation needs. The deterioration and acute exacerbation of the disease will disrupt the balance, thus damaging and further increasing the dysfunction of the diaphragm, which in severe cases may lead to respiratory failure and death. Diaphragm dysfunction mainly includes limitation of muscle strength, endurance, and mobility caused by many factors. Oxidative stress may affect diaphragm function in COPD through protein oxidation, protease activation, and decreased Ca^2+^ sensitivity. Exercise training can improve diaphragm dysfunction by regulating oxidative stress in COPD. Long-term (≥4 weeks) relatively high-intensity aerobic exercise seems to have a good effect on improving oxidative stress and dysfunction of the diaphragm in COPD. Compared with high-intensity exercise training, moderate-intensity (50–80% VO_2m__*ax*_) exercise training is more suitable for elderly patients. The mechanisms by which exercise influences oxidative stress in the diaphragm of patients with COPD may include increasing antioxidant activity, decreasing oxidase activity, and improving mitochondrial function. However, few studies have investigated the effect and mechanism of exercise on the oxidative stress level of COPD diaphragm. Thus, future studies should explore the effect and mechanism of different exercise prescriptions, including exercise style, intensity, and duration, on the oxidative stress level in the diaphragm of COPD. Furthermore, because the ultimate function of the COPD diaphragm depends on a special balance between injury and adaptation, more research related to the effects of exercise on this special balance should be conducted in the future.

## Author Contributions

WBW, XDL, and BZZ contributed to the design of this review. BZZ, JL, and PJL conducted the literature search selection and completed data extraction. BZZ completed data analysis and wrote the initial draft of the manuscript. PJL and JL contributed important intellectual content and put forward suggestions for revision. BZZ and PJL revised the article. WBW, XDL, and PJL supervised the quality of articles. All authors have read and approved the submitted version.

## Conflict of Interest

The authors declare that the research was conducted in the absence of any commercial or financial relationships that could be construed as a potential conflict of interest.

## References

[B1] Alcazar J. Losa-Reyna J. Rodriguez-Lopez C. Navarro-Cruz R. Alfaro-Acha A. Ara I. (2019). Effects of concurrent exercise training on muscle dysfunction and systemic oxidative stress in older people with COPD. *Scand. J. Med. Sci. Sports* 29 1591–1603. 10.1111/sms.13494 31169924

[B2] Barreiro E. Gea J. (2015). Respiratory and limb muscle dysfunction in COPD. *COPD* 12 413–426. 10.3109/15412555.2014.974737 25438125

[B3] Barreiro E. Peinado V. I. Galdiz J. B. Ferrer E. Marin-Corral J. Sánchez F. (2010). Cigarette smoke-induced oxidative stress: a role in chronic obstructive pulmonary disease skeletal muscle dysfunction. *Am. J. Respir. Crit. Care Med.* 182 477–488. 10.1164/rccm.200908-1220OC 20413628

[B4] Barreiro E. Salazar-Degracia A. Sancho-Muñoz A. Aguiló R. Rodríguez-Fuster A. Gea J. (2019). Endoplasmic reticulum stress and unfolded protein response in diaphragm muscle dysfunction of patients with stable chronic obstructive pulmonary disease. *J. Appl. Physiol. (1985)* 126 1572–1586. 10.1152/japplphysiol.00670.2018 30998124

[B5] Białas A. J. Sitarek P. Miłkowska-Dymanowska J. Piotrowski W. J. Górski P. (2016). The role of mitochondria and oxidative/antioxidative imbalance in pathobiology of chronic obstructive pulmonary disease. *Oxid. Med. Cell. Longev.* 2016:7808576. 10.1155/2016/7808576 28105251 PMC5220474

[B6] Borzone G. Zhao B. Merola A. J. Berliner L. Clanton T. L. (1994). Detection of free radicals by electron spin resonance in rat diaphragm after resistive loading. *J. Appl. Physiol. (1985)* 77 812–818. 10.1152/jappl.1994.77.2.812 8002533

[B7] Bouzid M. A. Filaire E. McCall A. Fabre C. (2015). Radical oxygen species, exercise and aging: an update. *Sports Med.* 45 1245–1261. 10.1007/s40279-015-0348-1 26119427

[B8] Bowen T. S. Aakerøy L. Eisenkolb S. Kunth P. Bakkerud F. Wohlwend M. (2017a). Exercise training reverses extrapulmonary impairments in smoke-exposed mice. *Med. Sci. Sports Exerc.* 49 879–887. 10.1249/MSS.0000000000001195 28009790

[B9] Bowen T. S. Eisenkolb S. Drobner J. Fischer T. Werner S. Linke A. (2017b). High-intensity interval training prevents oxidant-mediated diaphragm muscle weakness in hypertensive mice. *FASEB J.* 31 60–71. 10.1096/fj.201600672R 27650398

[B10] Bruells C. S. Marx G. (2018). [Diaphragm dysfunction : facts for clinicians]. *Med. Klin. Intensivmed. Notfmed.* 113 526–532. 10.1007/s00063-016-0226-0 27766377

[B11] Carlos S. P. Dias A. S. Forgiarini Júnior L. A. Patricio P. D. Graciano T. Nesi R. T. (2014). Oxidative damage induced by cigarette smoke exposure in mice: impact on lung tissue and diaphragm muscle. *J. Bras. Pneumol.* 40 411–420. 10.1590/s1806-37132014000400009 25210964 PMC4201172

[B12] Caron M. A. Debigaré R. Dekhuijzen P. N. R. Maltais F. (2009). Comparative assessment of the quadriceps and the diaphragm in patients with COPD. *J. Appl. Physiol. (1985)* 107 952–961. 10.1152/japplphysiol.00194.2009 19359618

[B13] Debigaré R. Côté C. H. Maltais F. (2010). Ubiquitination and proteolysis in limb and respiratory muscles of patients with chronic obstructive pulmonary disease. *Proc. Am. Thorac. Soc.* 7 84–90. 10.1513/pats.200906-051JS 20160153

[B14] Domínguez-Álvarez M. Sabaté-Brescó M. Vilà-Ubach M. Gáldiz J. B. Alvarez F. J. Casadevall C. (2014). Molecular and physiological events in respiratory muscles and blood of rats exposed to inspiratory threshold loading. *Transl. Res.* 163 478–493. 10.1016/j.trsl.2013.12.004 24373863

[B15] Donaldson A. V. Maddocks M. Martolini D. Polkey M. I. Man W. D. C. (2012). Muscle function in COPD: a complex interplay. *Int. J. Chron. Obstruct. Pulmon. Dis.* 7 523–535. 10.2147/COPD.S28247 22973093 PMC3430120

[B16] Dos Santos Yamaguti W. P. Paulin E. Shibao S. Chammas M. C. Salge J. M. Ribeiro M. (2008). Air trapping: the major factor limiting diaphragm mobility in chronic obstructive pulmonary disease patients. *Respirology* 13 138–144. 10.1111/j.1440-1843.2007.01194.x 18197925

[B17] Doucet M. Debigaré R. Joanisse D. R. Côté C. Leblanc P. Grégoire J. (2004). Adaptation of the diaphragm and the vastus lateralis in mild-to-moderate COPD. *Eur. Respir. J.* 24 971–979. 15572541 10.1183/09031936.04.00020204

[B18] Emtner M. Wadell K. (2016). Effects of exercise training in patients with chronic obstructive pulmonary disease–a narrative review for FYSS (Swedish Physical Activity Exercise Prescription Book). *Br. J. Sports Med.* 50 368–371. 10.1136/bjsports-2015-09587 26823440

[B19] Fang T. Wang M. Xiao H. Wei X. (2019). Mitochondrial dysfunction and chronic lung disease. *Cell Biol. Toxicol.* 35 493–502. 10.1007/s10565-019-09473-9 31119467

[B20] Gayan-Ramirez G. Decramer M. (2013). Mechanisms of striated muscle dysfunction during acute exacerbations of COPD. *J. Appl. Physiol. (1985)* 114 1291–1299. 10.1152/japplphysiol.00847.2012 23372146

[B21] Gea J. Agustí A. Roca J. (2013). Pathophysiology of muscle dysfunction in COPD. *J. Appl. Physiol. (1985)* 114 1222–1234. 10.1152/japplphysiol.00981.2012 23519228

[B22] Gimeno-Santos E. Rodriguez D. A. Barberan-Garcia A. Blanco I. Vilaró J. Torralba Y. (2014). Endurance exercise training improves heart rate recovery in patients with COPD. *COPD* 11 190–196. 10.3109/15412555.2013.831401 24377907

[B23] Gomes-Marcondes M. C. C. Tisdale M. J. (2002). Induction of protein catabolism and the ubiquitin-proteasome pathway by mild oxidative stress. *Cancer Lett.* 180 69–74. 10.1016/s0304-3835(02)00006-x 11911972

[B24] Gonçalves M. A. Leal B. E. Lisboa L. G. Tavares M. G. S. Yamaguti W. P. Paulin E. (2018). Comparison of diaphragmatic mobility between COPD patients with and without thoracic hyperkyphosis: a cross-sectional study. *J. Bras. Pneumol.* 44 5–11. 10.1590/S1806-37562016000000248 29538536 PMC6104535

[B25] Gosker H. R. Wouters E. F. van der Vusse G. J. Schols A. M. (2000). Skeletal muscle dysfunction in chronic obstructive pulmonary disease and chronic heart failure: underlying mechanisms and therapy perspectives. *Am. J. Clin. Nutr.* 71 1033–1047. 10.1093/ajcn/71.5.1033 10799364

[B26] Gu Q. Wang B. Zhang X.-F. Ma Y.-P. Liu J.-D. Wang X.-Z. (2014). Chronic aerobic exercise training attenuates aortic stiffening and endothelial dysfunction through preserving aortic mitochondrial function in aged rats. *Exp. Gerontol.* 56 37–44. 10.1016/j.exger.2014.02.014 24607516

[B27] Hasan-Olive M. M. Lauritzen K. H. Ali M. Rasmussen L. J. Storm-Mathisen J. Bergersen L. H. (2019). A ketogenic diet improves mitochondrial biogenesis and bioenergetics via the PGC1α-SIRT3-UCP2 axis. *Neurochem. Res.* 44 22–37. 10.1007/s11064-018-2588-6 30027365

[B28] Herzig S. Shaw R. J. (2018). AMPK: guardian of metabolism and mitochondrial homeostasis. *Nat. Rev. Mol. Cell. Biol.* 19 121–135. 10.1038/nrm.2017.95 28974774 PMC5780224

[B29] Heunks L. M. Dekhuijzen P. N. (2000). Respiratory muscle function and free radicals: from cell to COPD. *Thorax* 55 704–716. 10.1136/thorax.55.8.704 10899251 PMC1745815

[B30] Heunks L. M. Bast A. van Herwaarden C. L. Haenen G. R. Dekhuijzen P. N. (2000). Effects of emphysema and training on glutathione oxidation in the hamster diaphragm. *J. Appl. Physiol. (1985)* 88 2054–2061. 10.1152/jappl.2000.88.6.2054 10846018

[B31] Heunks L. M. Cody M. J. Geiger P. C. Dekhuijzen P. N. Sieck G. C. (2001). Nitric oxide impairs Ca2+ activation and slows cross-bridge cycling kinetics in skeletal muscle. *J. Appl. Physiol. (1985)* 91 2233–2239. 10.1152/jappl.2001.91.5.2233 11641366

[B32] Itoh M. Oh-Ishi S. Hatao H. Leeuwenburgh C. Selman C. Ohno H. (2004). Effects of dietary calcium restriction and acute exercise on the antioxidant enzyme system and oxidative stress in rat diaphragm. *Am. J. Physiol. Regul. Integr. Comp. Physiol.* 287 R33–R38. 10.1152/ajpregu.00598.2003 14764436

[B33] Ji L. L. Leeuwenburgh C. Leichtweis S. Gore M. Fiebig R. Hollander J. (1998). Oxidative stress and aging. Role of exercise and its influences on antioxidant systems. *Ann. N. Y. Acad. Sci.* 854 102–117. 10.1111/j.1749-6632.1998.tb09896.x 9928424

[B34] Jia X. L. (2011). Research progress of UCP2. *J. Chifeng Univ.* 27 39–41. 10.3969/j.issn.1673-260X.2011.07.018

[B35] Klimathianaki M. Vaporidi K. Georgopoulos D. (2011). Respiratory muscle dysfunction in COPD: from muscles to cell. *Curr. Drug Targets* 12 478–488. 10.2174/138945011794751474 21194407

[B36] Kriegenburg F. Poulsen E. G. Koch A. Krüger E. Hartmann-Petersen R. (2011). Redox control of the ubiquitin-proteasome system: from molecular mechanisms to functional significance. *Antioxid. Redox Signal.* 15 2265–2299. 10.1089/ars.2010.3590 21314436

[B37] Langen R. C. J. Korn S. H. Wouters E. F. M. (2003). ROS in the local and systemic pathogenesis of COPD. *Free. Radic. Biol. Med.* 35 226–235. 10.1016/s0891-5849(03)00316-2 12885585

[B38] Levine S. Bashir M. H. Clanton T. L. Powers S. K. Singhal S. (2013). COPD elicits remodeling of the diaphragm and vastus lateralis muscles in humans. *J. Appl. Physiol. (1985)* 114 1235–1245. 10.1152/japplphysiol.01121.2012 23264538 PMC3656432

[B39] Levine S. Kaiser L. Leferovich J. Tikunov B. (1997). Cellular adaptations in the diaphragm in chronic obstructive pulmonary disease. *N. Engl. J. Med.* 337 1799–1806. 10.1056/NEJM199712183372503 9400036

[B40] Levine S. Nguyen T. Kaiser L. R. Rubinstein N. A. Maislin G. Gregory C. (2003). Human diaphragm remodeling associated with chronic obstructive pulmonary disease: clinical implications. *Am. J. Respir. Crit. Care Med.* 168 706–713. 10.1164/rccm.200209-1070OC 12857719

[B41] Li H. Duan H. J. (2011). Nrf2/ARE pathway and downstream antioxidant genes. *Chin. Pharmacol. Bull.* 27 300–303.

[B42] Li J. Lu Y. Li N. Li P. Su J. Wang Z. (2020). Muscle metabolomics analysis reveals potential biomarkers of exercise-dependent improvement of the diaphragm function in chronic obstructive pulmonary disease. *Int. J. Mol. Med.* 45 1644–1660. 10.3892/ijmm.2020.4537 32186768 PMC7169662

[B43] Lin J.-Y. Kuo W.-W. Baskaran R. Kuo C.-H. Chen Y.-A. Chen W. S.-T. (2020). Swimming exercise stimulates IGF1/PI3K/Akt and AMPK/SIRT1/PGC1α survival signaling to suppress apoptosis and inflammation in aging hippocampus. *Aging* 12 6852–6864. 10.18632/aging.103046 32320382 PMC7202519

[B44] Liu D. J. Han X. P. Song Y. Y. Liu J. B. (2017). Expression of calpains and calpastatin in diaphragm of a rat model of COPD. *J. Prac. Med.* 33 1754–1756.

[B45] Lopes A. J. Vigário P. S. Hora A. L. Deus C. A. Soares M. S. Guimaraes F. S. (2018). Ventilation distribution, pulmonary diffusion and peripheral muscle endurance as determinants of exercise intolerance in elderly patients with chronic obstructive pulmonary disease. *Physiol. Res.* 67 863–874. 10.33549/physiolres.933867 30204461

[B46] López-Campos J. L. Tan W. Soriano J. B. (2016). Global burden of COPD. *Respirology* 21 14–23. 10.1111/resp.12660 26494423

[B47] Macnee W. (2001). Oxidative stress and lung inflammation in airway disease. *Eur. J. Pharmacol.* 429 195–207. 10.1016/S0014-2999(01)01320-6 11698041

[B48] Mangner N. Bowen T. S. Werner S. Fischer T. Kullnick Y. Oberbach A. (2016). Exercise training prevents diaphragm contractile dysfunction in heart failure. *Med. Sci. Sports Exerc.* 48 2118–2124. 10.1249/MSS.0000000000001016 27327028

[B49] Mangner N. Linke A. Oberbach A. Kullnick Y. Gielen S. Sandri M. (2013). Exercise training prevents TNF-α induced loss of force in the diaphragm of mice. *PLoS One* 8:e52274. 10.1371/journal.pone.0052274 23300968 PMC3534708

[B50] Marin-Corral J. Minguella J. Ramírez-Sarmiento A. L. Hussain S. N. A. Gea J. Barreiro E. (2009). Oxidised proteins and superoxide anion production in the diaphragm of severe COPD patients. *Eur. Respir. J.* 33 1309–1319. 10.1183/09031936.00072008 19196822

[B51] Nakatani K. Komatsu M. Kato T. Yamanaka T. Takekura H. Wagatsuma A. (2005). Habitual exercise induced resistance to oxidative stress. *Free Radic. Res.* 39 905–911. 16087471 10.1080/10715760500183300

[B52] Nici L. Donner C. Wouters E. Zuwallack R. Ambrosino N. Bourbeau J. (2006). American Thoracic Society/European Respiratory Society statement on pulmonary rehabilitation. *Am. J. Respir. Crit. Care Med.* 173 1390–1413. 10.1164/rccm.200508-1211ST 16760357

[B53] Oh-ishi S. Kizaki T. Ookawara T. Sakurai T. Izawa T. Nagata N. (1997). Endurance training improves the resistance of rat diaphragm to exercise-induced oxidative stress. *Am. J. Respir. Crit. Care Med.* 156 1579–1585. 10.1164/ajrccm.156.5.96-11035 9372679

[B54] Orozco-Levi M. (2003). Structure and function of the respiratory muscles in patients with COPD: impairment or adaptation? *Eur. Respir. J. Suppl.* 46 41s–51s. 10.1183/09031936.03.00004607 14621106

[B55] Orozco-Levi M. Gea J. Lloreta J. L. Félez M. Minguella J. Serrano S. (1999). Subcellular adaptation of the human diaphragm in chronic obstructive pulmonary disease. *Eur. Respir. J.* 13 371–378. 10.1183/09031936.99.13237199 10065684

[B56] Orozco-Levi M. Lloreta J. Minguella J. Serrano S. Broquetas J. M. Gea J. (2001). Injury of the human diaphragm associated with exertion and chronic obstructive pulmonary disease. *Am. J. Respir. Crit. Care Med.* 164 1734–1739. 10.1164/ajrccm.164.9.2011150 11719318

[B57] Ottenheijm C. A. C. Heunks L. M. A. Dekhuijzen P. N. R. (2007). Diaphragm muscle fiber dysfunction in chronic obstructive pulmonary disease: toward a pathophysiological concept. *Am. J. Respir. Crit. Care Med.* 175 1233–1240. 10.1164/rccm.200701-020PP 17413128

[B58] Ottenheijm C. A. C. Heunks L. M. A. Dekhuijzen R. P. N. (2008). Diaphragm adaptations in patients with COPD. *Respir. Res.* 9:12. 10.1186/1465-9921-9-12 18218129 PMC2248576

[B59] Ottenheijm C. A. C. Heunks L. M. A. Li Y. P. Jin B. Minnaard R. van Hees H. W. H. (2006). Activation of the ubiquitin-proteasome pathway in the diaphragm in chronic obstructive pulmonary disease. *Am. J. Respir. Crit. Care Med.* 174 997–1002. 10.1164/rccm.200605-721OC 16917114 PMC2648103

[B60] Ottenheijm C. A. C. Heunks L. M. A. Sieck G. C. Zhan W. Z. Jansen S. M. Degens H. (2005). Diaphragm dysfunction in chronic obstructive pulmonary disease. *Am. J. Respir. Crit. Care Med.* 172 200–205. 10.1164/rccm.200502-262OC 15849324 PMC2718467

[B61] Paneroni M. Simonelli C. Vitacca M. Ambrosino N. (2017). Aerobic exercise training in very severe chronic obstructive pulmonary disease: a systematic review and meta-analysis. *Am. J. Phys. Med. Rehabil.* 96 541–548. 10.1097/PHM.0000000000000667 28099192

[B62] Paulin E. Yamaguti W. P. S. Chammas M. C. Shibao S. Stelmach R. Cukier A. (2007). Influence of diaphragmatic mobility on exercise tolerance and dyspnea in patients with COPD. *Respir. Med.* 101 2113–2118. 10.1016/j.rmed.2007.05.024 17644365

[B63] Polkey M. I. Kyroussis D. Hamnegard C. H. Mills G. H. Green M. Moxham J. (1996). Diaphragm strength in chronic obstructive pulmonary disease. *Am. J. Respir. Crit. Care Med.* 154 1310–1317. 10.1164/ajrccm.154.5.8912741 8912741

[B64] Pomiès P. Blaquière M. Maury J. Mercier J. Gouzi F. Hayot M. (2016). Involvement of the FoxO1/MuRF1/atrogin-1 signaling pathway in the oxidative stress-induced atrophy of cultured chronic obstructive pulmonary disease myotubes. *PLoS One* 11:e0160092. 10.1371/journal.pone.0160092 27526027 PMC4987766

[B65] Powers S. K. Jackson M. J. (2008). Exercise-induced oxidative stress: cellular mechanisms and impact on muscle force production. *Physiol. Rev.* 88 1243–1276. 10.1152/physrev.00031.2007 18923182 PMC2909187

[B66] Raherison C. Girodet P. O. (2009). Epidemiology of COPD. *Eur. Respir. Rev.* 18 213–221. 10.1183/09059180.00003609 20956146

[B67] Ribera F. N’Guessan B. Zoll J. Fortin D. Serrurier B. Mettauer B. (2003). Mitochondrial electron transport chain function is enhanced in inspiratory muscles of patients with chronic obstructive pulmonary disease. *Am. J. Respir. Crit. Care Med.* 167 873–879. 10.1164/rccm.200206-519OC 12493645

[B68] Ries A. L. Bauldoff G. S. Carlin B. W. Casaburi R. Emery C. F. Mahler D. A. (2007). Pulmonary rehabilitation: joint ACCP/AACVPR evidence-based clinical practice guidelines. *Chest* 131(Suppl. 5) 4S–42S. 10.1378/chest.06-2418 17494825

[B69] Rocha F. R. Brüggemann A. K. V. Francisco D. S. Medeiros C. S. Rosal D. Paulin E. (2017). Diaphragmatic mobility: relationship with lung function, respiratory muscle strength, dyspnea, and physical activity in daily life in patients with COPD. *J. Bras. Pneumol.* 43 32–37. 10.1590/S1806-37562016000000097 28380186 PMC5790674

[B70] Rubattu S. Stanzione R. Volpe M. (2016). Mitochondrial dysfunction contributes to hypertensive target organ damage: lessons from an animal model of human disease. *Oxid. Med. Cell Longev.* 2016:1067801. 10.1155/2016/1067801 27594970 PMC4993945

[B71] Schultz K. Jelusic D. Wittmann M. Krämer B. Huber V. Fuchs S. (2018). Inspiratory muscle training does not improve clinical outcomes in 3-week COPD rehabilitation: results from a randomised controlled trial. *Eur. Respir. J.* 51:1702000. 10.1183/13993003.02000-2017 29371382

[B72] Sheng H. Zhang Y. Shi X. Hu Y. Pang B. Jin J. (2020). Functional, ultrastructural, and transcriptomic changes in rat diaphragms with different durations of cigarette smoke exposure. *Int. J. Chron. Obstruct. Pulmon. Dis.* 15 3135–3145. 10.2147/COPD.S278327 33299306 PMC7721115

[B73] Similowski T. Yan S. Gauthier A. P. Macklem P. T. Bellemare F. (1991). Contractile properties of the human diaphragm during chronic hyperinflation. *N. Engl. J. Med.* 325 917–923. 10.1056/NEJM199109263251304 1881417

[B74] Singh D. Agusti A. Anzueto A. Barnes P. J. Bourbeau J. Celli B. R. (2019). Global Strategy for the Diagnosis, Management, and Prevention of Chronic Obstructive Lung Disease: the GOLD science committee report 2019. *Eur. Respir. J.* 53:1900164. 10.1183/13993003.00164-2019 30846476

[B75] Smuder A. J. Min K. Hudson M. B. Kavazis A. N. Kwon O. S. Nelson W. B. (2012). Endurance exercise attenuates ventilator-induced diaphragm dysfunction. *J. Appl. Physiol. (1985)* 112 501–510. 10.1152/japplphysiol.01086.2011 22074717 PMC4587591

[B76] Spruit M. A. Singh S. J. Garvey C. ZuWallack R. Nici L. Rochester C. (2013). An official American Thoracic Society/European Respiratory Society statement: key concepts and advances in pulmonary rehabilitation. *Am. J. Respir. Crit. Care Med.* 188 e13–e64. 10.1164/rccm.201309-1634ST 24127811

[B77] Vieira Ramos G. Choqueta de Toledo-Arruda A. Maria Pinheiro-Dardis C. Liyoko Suehiro C. Luiz de Russo T. Vieira R. P. (2018). Exercise prevents diaphragm wasting induced by cigarette smoke through modulation of antioxidant genes and metalloproteinases. *Biomed Res. Int.* 2018:5909053. 10.1155/2018/5909053 29789801 PMC5896353

[B78] Vilaró J. Ramirez-Sarmiento A. Martínez-Llorens J. M. A. Mendoza T. Alvarez M. Sánchez-Cayado N. (2010). Global muscle dysfunction as a risk factor of readmission to hospital due to COPD exacerbations. *Respir. Med.* 104 1896–1902. 10.1016/j.rmed.2010.05.001 20541383

[B79] Vincent H. K. Powers S. K. Stewart D. J. Demirel H. A. Shanely R. A. Naito H. (2000). Short-term exercise training improves diaphragm antioxidant capacity and endurance. *Eur. J. Appl. Physiol.* 81 67–74. 10552269 10.1007/PL00013799

[B80] Vincent K. R. Vincent H. K. Braith R. W. Lennon S. L. Lowenthal D. T. (2002). Resistance exercise training attenuates exercise-induced lipid peroxidation in the elderly. *Eur. J. Appl. Physiol.* 87 416–423. 10.1007/s00421-002-0640-2 12172882

[B81] Whidden M. A. Smuder A. J. Wu M. Hudson M. B. Nelson W. B. Powers S. K. (2010). Oxidative stress is required for mechanical ventilation-induced protease activation in the diaphragm. *J. Appl. Physiol. (1985)* 108 1376–1382. 10.1152/japplphysiol.00098.2010 20203072 PMC2867537

[B82] Wieczfinska J. Sitarek P. Skała E. Kowalczyk T. Pawliczak R. (2019). Inhibition of NADPH oxidase-derived reactive oxygen species decreases expression of inflammatory cytokines in A549 cells. *Inflammation* 42 2205–2214. 10.1007/s10753-019-01084-0 31612365 PMC6856491

[B83] Wijnhoven J. H. Janssen A. J. M. van Kuppevelt T. H. Rodenburg R. J. T. Dekhuijzen P. N. R. (2006). Metabolic capacity of the diaphragm in patients with COPD. *Respir. Med.* 100 1064–1071. 10.1016/j.rmed.2005.09.029 16257195

[B84] Xie Z. Zhang J. Wu J. Viollet B. Zou M. H. (2008). Upregulation of mitochondrial uncoupling protein-2 by the AMP-activated protein kinase in endothelial cells attenuates oxidative stress in diabetes. *Diabetes* 57 3222–3230. 10.2337/db08-0610 18835932 PMC2584127

[B85] Zhou X. Yi D. Wu Y. Pei X. Yu H. Chen Y. (2018). Expression of diaphragmatic myostatin and correlation with apoptosis in rats with chronic obstructive pulmonary disease. *Exp. Ther. Med.* 15 2295–2300. 10.3892/etm.2018.5686 29456636 PMC5795556

